# Identification of a 6‐lncRNA prognostic signature based on microarray re‐annotation in gastric cancer

**DOI:** 10.1002/cam4.2621

**Published:** 2019-11-19

**Authors:** Bin Ma, Yongmin Li, Yupeng Ren

**Affiliations:** ^1^ Department of Colorectal Surgery Cancer Hospital of China Medical University Liaoning Cancer Hospital and Institute Shenyang Liaoning Province People's Republic of China

**Keywords:** gastric cancer, GEO, least absolute shrinkage and selection operator (LASSO), long noncoding RNAs, prognosis, robust likelihood‐based survival

## Abstract

Gastric cancer (GC) remains an important malignancy worldwide with poor prognosis. Long noncoding RNAs (lncRNAs) can markedly affect cancer progression. Moreover, lncRNAs have been proposed as diagnostic or prognostic biomarkers of GC. Therefore, the current study aimed to explore lncRNA‐based prognostic biomarkers for GC. LncRNA expression profiles from the Gene Expression Omnibus (GEO) database were first downloaded. After re‐annotation of lncRNAs, a univariate Cox analysis identified 177 prognostic lncRNA probes in the training set http://www.ncbi.nlm.nih.gov/geo/query/acc.cgi?acc=GSE62254 (n = 225). Multivariate Cox analysis of each lncRNA with clinical characteristics as covariates identified a total of 46 prognostic lncRNA probes. Robust likelihood‐based survival and least absolute shrinkage and selection operator (LASSO) models were used to establish a 6‐lncRNA signature with prognostic value. Receiver operating characteristic (ROC) curve analyses were employed to compare survival prediction in terms of specificity and sensitivity. Patients with high‐risk scores exhibited a significantly worse overall survival (OS) than patients with low‐risk scores (log‐rank test *P*‐value <.0001), and the area under the ROC curve (AUC) for 5‐year survival was 0.77. A nomogram and forest plot were constructed to compare the clinical characteristics and risk scores by a multivariable Cox regression analysis, which suggested that the 6‐lncRNA signature can independently make the prognosis evaluation of patients. Single‐sample GSEA (ssGSEA) was used to determine the relationships between the 6‐lncRNA signature and biological functions. The internal validation set http://www.ncbi.nlm.nih.gov/geo/query/acc.cgi?acc=GSE62254 (n = 75) and the external validation set http://www.ncbi.nlm.nih.gov/geo/query/acc.cgi?acc=GSE57303 (n = 70) were successfully used to validate the robustness of our 6‐lncRNA signature. In conclusion, based on the above results, the 6‐lncRNA signature can effectively make the prognosis evaluation of GC patients.

## Graphical abstract

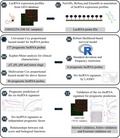

## 1 INTRODUCTION

Gastric cancer (GC) is an important cancer worldwide, and nearly 1 000 000 new cases were reported in 2018. GC is a common cancer (ranking as the fifth) and the third common cause of cancer death.^1^ The incidence of GC morbidity is two times higher in males than that in females. *Helicobacter pylori* has been shown to be the major risk factor for GC, and the new cases caused by *H pylori* infection account for nearly 90% of all GC cases.^2^, ^3^ Surgical treatment remains the first line of treatment for GC patients. However, despite the advances in surgical methods, radiotherapy, chemotherapy, and neoadjuvant therapy, the prognosis of GC remains poor.^4^ GC prognosis highly varies by various regions, with an estimated 783 000 deaths in 2018.^1^ Thus, reducing the incidence of GC remains the key to reducing mortality.^5^, ^6^ Because this cancer results in a 20%‐30% survival rate at 5 years and a 5%‐10% survival rate in advanced stages,^7^ the clinical outcomes of patients are unsatisfactory. Therefore, the goal of the current study was to explore prognostic evaluation biomarkers in GC.

Long noncoding RNA (lncRNAs) are transcripts with more than 200 nucleotides, and dysregulated lncRNAs are associated with various human diseases.^8^ It has been reported that lncRNAs control several biological processes that affect multiple levels of gene expression from transcription to protein localization and stabilization.^9^ LncRNAs are commonly reported in human cancers including GC,^10^ breast,^11^ bladder,^12^ colon,^13^ and other cancers. LncRNAs can play important roles in cancer progression‐associated pathways, such as proliferation, growth, migration, invasion, and apoptosis. Many well‐known lncRNAs, such as HOTAIR,^14^ H19,^15^ BLACAT1,^16^ PCAT‐1,^17^ MEG3,^18^ and MALAT1,^19^ have been shown to be oncogenic factors or tumor suppressors. Additionally, these lncRNAs have been proposed as diagnostic or prognostic biomarkers of GC.

Recently, based on microarray and RNA‐seq methods, together with available open databases such as The Cancer Genome Atlas (TCGA) and Gene Expression Omnibus (GEO), people can easily obtain expression data for human cancers. In addition, using bioinformatics analysis, lncRNA signatures with prognostic value were established. For example, a two‐lncRNA signature, a potential biomarker for the prognosis of cervical cancer, was identified using a public database.^20^ Furthermore, there are different survival‐related lncRNA signatures in hepatocellular carcinoma,^21^ lung cancer,^22^ and pancreatic cancer.^23^ However, there have been few related studies in GC that can provide us with relevant insights.

In this study, we performed a multistep re‐annotation analysis of lncRNA expression in GC. Based on lncRNA expression profiles in http://www.ncbi.nlm.nih.gov/geo/query/acc.cgi?acc=GSE62254, we used robust likelihood‐based survival and LASSO models to establish a 6‐lncRNA signature with prognostic value. Patients with high‐risk scores had markedly worse overall survival (OS) than patients with low‐risk scores (log‐rank test *P*‐value <.0001), and the AUC for 5‐year survival was 0.77. In addition, the 6‐lncRNA signature could be used to independently make the prognosis evaluation of GC patients. The internal validation set http://www.ncbi.nlm.nih.gov/geo/query/acc.cgi?acc=GSE62254 and the external validation set http://www.ncbi.nlm.nih.gov/geo/query/acc.cgi?acc=GSE57303 were successfully used to validate the robustness of our 6‐lncRNA signature. In conclusion, based on the above results, the 6‐lncRNA signature displays a strong power for prognostic evaluation in GC patients.

## 2 MATERIALS AND METHODS

### 2.1 Data downloading and processing

We downloaded gene expression profiles of GC samples as MINiML formatted family files from the GEO database, which are available athttps://www.ncbi.nlm.nih.gov/geo/.^24^ Here, a total of two GEO datasets, namely, http://www.ncbi.nlm.nih.gov/geo/query/acc.cgi?acc=GSE62254 25 and http://www.ncbi.nlm.nih.gov/geo/query/acc.cgi?acc=GSE57303,^26^ were selected. The http://www.ncbi.nlm.nih.gov/geo/query/acc.cgi?acc=GSE62254 dataset contains 300 GC samples with clinical information (Table S1). In addition, http://www.ncbi.nlm.nih.gov/geo/query/acc.cgi?acc=GSE57303, used as an external validation set, contains 70 GC samples with clinical information (Table S2). Both used the Affymetrix Human Genome U133 Plus 2.0 Array platform. The clinical data included pathological T, N, and M classification, stage, and survival information of GC patients.

### 2.2 Re‐annotation of lncRNA classification

To evaluate lncRNA expression based on probe ID, we used the methods described by Zhang et al.^27^ Briefly,

1. We first mapped the Affymetrix Human Genome U133 Plus 2.0 Array probe set ID to NetAffx Annotation Files (http://www.affymetrix.com). The NetAffx files are the direct platform for probes including the probe set ID, gene symbol, gene title, Ensembl gene ID, Refseq transcript ID, and other information for the specific probe set.
2. Second, we extracted the probe sets assigned with an Ensembl gene ID and/or Refseq transcript ID in NetAffx annotations.
3. Then, for Refseq transcript ID, we only retained those labeled as “NR_” (NR represents nonprotein‐coding transcript in NCBI Reference Sequence Database). The short noncoding RNAs such as pseudogenes, microRNAs, and other short RNAs were removed.
4. For the probe sets with Ensembl IDs, we only retained those annotated with “lincRNA,” “processed transcripts,” “non‐coding,” or “misc_RNA” in Ensembl annotations.
5. Finally, corresponding Affymetrix probe IDs were used to generate annotated lncRNA transcript profiles (Table S3).

### 2.3 Univariate and multivariate Cox proportional hazard model

To select key lncRNA probes with prognostic values, we divided all patients in http://www.ncbi.nlm.nih.gov/geo/query/acc.cgi?acc=GSE62254 into two sets, namely, (a) a training set and (b) an internal validation set, in a random manner according to a ratio of 3:1. Next, we used the expression profiles of the above re‐annotated lncRNA probes and patients in the training set (n = 225). Using the R package *survival*, we performed a univariate Cox proportional hazards analysis using the *coxph* function. The statistical significance cutoff of the *P*‐value was considered at <.01. Then, we assessed other clinical features, including age, pT, pN, pM, and pStage, by Kaplan‐Meier analyses. The statistical significance cutoff of the *P*‐value was the same as above. Finally, we considered the above clinical factors as covariates. We performed a multivariate Cox proportional hazards analysis using prognostic lncRNA probes combined with clinical factors.

### 2.4 Prognosis‐related lncRNA selection by a robust likelihood‐based survival model

Robust likelihood‐based survival modeling was carried out for selecting prognosis‐related lncRNAs using the R package *rbsurv*. This model uses the partial likelihood of the Cox model as the underlying method. For robustness, this package selects survival‐associated genes by separating the two sets as a cross‐validation technology with large variability. It employs forward selection, generating some gene models, and selecting an optimal model by Akaike information criteria (AIC). According to the study by Wang et al,^28^ briefly, we randomly selected 75% of all samples in the training set using threefold cross‐validation. Moreover, the maximum number of genes was set to 30, and the analysis was repeated 1000 times. Finally, we summarized the results of each dimensionality reduction.

### 2.5 Construction of a prognostic lncRNA signature by LASSO modeling

Based on the above identification of prognosis‐related lncRNA probes for GC, we further needed to narrow the gene range and establish a prognostic signature. Thus, we performed LASSO analysis, which constructs a more refined model using a penalty function. This method can compress some coefficients to zero; therefore, some unimportant indicators are reduced to 0, leaving a small number of indicators, for which the weight is not 0. LASSO analysis was implemented with the R package *glmnet*.^29^

### 2.6 Prognostic evaluation using the 6‐lncRNA signature

Each lncRNA probe was accompanied by a formula of risk score, and the estimated regression coefficients were used to weight the formula in LASSO analysis. GC patients in the training and validation sets were divided into two groups, a group with high risk and a group with low risk, by taking the corresponding median risk score as the cutoff point. Kaplan‐Meier curves were used to compare the two groups regarding their survival outcomes with the assistance of the log‐rank test. Receiver operating characteristic (ROC) curve analysis was employed to compare survival prediction with regard to the specificity and sensitivity according to lncRNA risk scores. A *P*‐value <.05 was considered significant.

### 2.7 Analysis of the 6‐lncRNA signature and clinical characteristics

To examine the relationship between the prediction accuracy of prognostic signature and clinical characteristics such as age, sex, pStage, pT, pN, pM, and risk scores, we employed a univariate Cox proportional hazards model. Meanwhile, the differential patterns of various clinical characteristics were analyzed. A nomogram and forest plot were used to display the results of the multivariable Cox analysis including all of the above variables. The R packages *rms* and *forestplot* were used to construct the nomogram and forest plot, respectively.

### 2.8 Single‐sample GSEA (ssGSEA)

To observe the relationship between risk scores and biological functions, we used ssGSEA^30^ using the R package GSVA. ssGSEA, also called Gene Set Variation Analysis (GSVA), is a special Gene Set Enrichment Analysis (GSEA) that is mainly used for GSEA using single samples. This analysis enables the robust identification of a detailed change in pathway activity in a sample. Pearson's analysis was used to identify the correlation between risk scores and pathways. The cutoff of correlation was set at 0.3.

### 2.9 Validation of the 6‐lncRNA signature

The same risk formula was used to validate the internal validation set http://www.ncbi.nlm.nih.gov/geo/query/acc.cgi?acc=GSE62254 (n = 75), the entire set http://www.ncbi.nlm.nih.gov/geo/query/acc.cgi?acc=GSE62254 (n = 300), and the external validation set http://www.ncbi.nlm.nih.gov/geo/query/acc.cgi?acc=GSE57303 (n = 70). The Kaplan‐Meier curves for OS were used to compare the two groups regarding survival outcomes with the log‐rank test.

## 3 RESULTS

### 3.1 Prognosis‐related lncRNA screening by univariate Cox proportional hazard modeling

**Figure 1:**
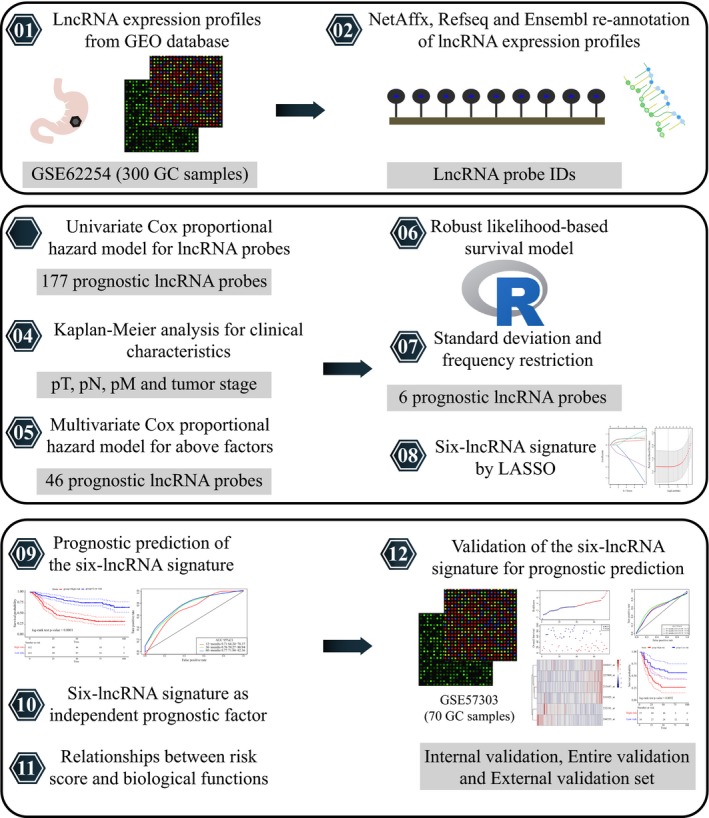
The schematic workflow of the present study

In this study, we performed a multistep re‐annotation analysis of lncRNA expression in GC (Figure 1). Based on the NetAffx Annotation Files (Ensembl gene ID and/or Refseq transcript ID) and above re‐annotation methods, a total of 2448 probe IDs (corresponding to 1970 lncRNA genes) were determined. The Ensembl and Refseq databases contributed to the annotation of 725 probe IDs (510 genes) in common. Besides, 512 probe IDs (379 genes) were annotated only by the Refseq database, and 1211 probe IDs (1081 genes) were annotated only by the Ensembl database.

To select key lncRNA features with prognostic values, we employed a univariate Cox proportional hazards model. The expression data and clinical information corresponding to the http://www.ncbi.nlm.nih.gov/geo/query/acc.cgi?acc=GSE62254 dataset were first obtained. The samples in http://www.ncbi.nlm.nih.gov/geo/query/acc.cgi?acc=GSE62254 were divided into two sets based on the ratio of 3:1 in a random manner: the first is a training set, and the other is an internal validation set. The two sets contained 225 and 75 GC samples, respectively. http://www.ncbi.nlm.nih.gov/geo/query/acc.cgi?acc=GSE57303 (n = 70) was used as an external validation set. We included the patient's age, survival status, sex, T stage, N stage, M stage, and tumor stage (Table 1). A univariate Cox proportional hazards regression model was employed for 2448 re‐annotated lncRNAs and survival data in the training set. The *coxph* function in the R package *survival* was used to finally identify 177 prognostic lncRNA probes with a cutoff *P*‐value <.01 (Table S4). The most significant of the top 20 lncRNA probes are shown in Table 2.

**Table 1:** The demographic characteristics of samples in the training and validation datasets

Characteristics	Training dataset http://www.ncbi.nlm.nih.gov/geo/query/acc.cgi?acc=GSE62254 (n = 225)	Validation dataset http://www.ncbi.nlm.nih.gov/geo/query/acc.cgi?acc=GSE62254 (n = 75)	Validation dataset http://www.ncbi.nlm.nih.gov/geo/query/acc.cgi?acc=GSE57303 (n = 70)
Age (y)			
≤60	87	30	29
>60	138	45	39
Survival status			
Living	116	32	34
Dead	109	43	36
Gender			
Female	70	31	18
Male	155	44	52
pT			
T2	143	43	7
T3	67	24	54
T4	13	8	9
pN			
N0	24	14	13
N1	103	28	26
N2	63	17	26
N3	35	16	5
pM			
M0	202	68	63
M1	20	7	7
pStage			
Stage I	20	10	3
Stage II	77	19	9
Stage III	74	21	41
Stage IV	52	25	17
Lauren subtype			
Diffuse	107	35	35
Intestinal	112	38	20
Mixed	6	2	15
MLH1 IHC			
Negative	48	16	—
Positive	176	58	—
EBV ISH			
Negative	192	65	—
Positive	15	3	—
Molecular subtype			
MSS/TP53‐	82	25	—
MSS/TP53+	59	20	—
MSI	48	20	—
EMT	36	10	—

Abbreviations: pM, pathology Metastasis stage; pN, pathology Lymph Node stage; pT, pathology Tumor stage.

**Table 2:** The most significant of the top 20 lncRNA probes by univariate Cox proportional hazard model

Probe IDs	*P*‐value	HR	Low 95% CI	High 95% CI
236141_at	1.08E−07	2.656	1.853	3.809
213447_at	2.12E−07	4.327	2.488	7.525
219791_s_at	4.61E−07	3.118	2.004	4.851
1559901_s_at	1.31E−06	11.148	4.198	29.602
1564139_at	2.01E−06	7.717	3.322	17.925
221974_at	3.03E−06	2.612	1.745	3.908
235759_at	3.49E−06	2.345	1.636	3.362
227909_at	4.17E−06	4.957	2.507	9.801
242358_at	4.65E−06	4.243	2.286	7.876
226582_at	7.22E−06	2.511	1.679	3.753
1558828_s_at	7.35E−06	2.647	1.730	4.052
1556695_a_at	7.90E−06	6.592	2.882	15.075
229734_at	7.98E−06	12.126	4.056	36.254
230589_at	9.05E−06	27.102	6.313	116.348
1559965_at	9.98E−06	11.411	3.874	33.606
232298_at	1.10E−05	2.196	1.546	3.119
244553_at	1.33E−05	0.070	0.021	0.232
1556364_at	1.34E−05	5.857	2.643	12.978
225381_at	1.34E−05	2.108	1.507	2.949
241834_at	1.86E−05	9.697	3.427	27.433

Abbreviations: HR: hazard ratio; CI: confidence interval.

### 3.2 Prognosis‐related lncRNA screening by multivariate Cox proportional hazards modeling with clinical characteristics

**Figure 2:**
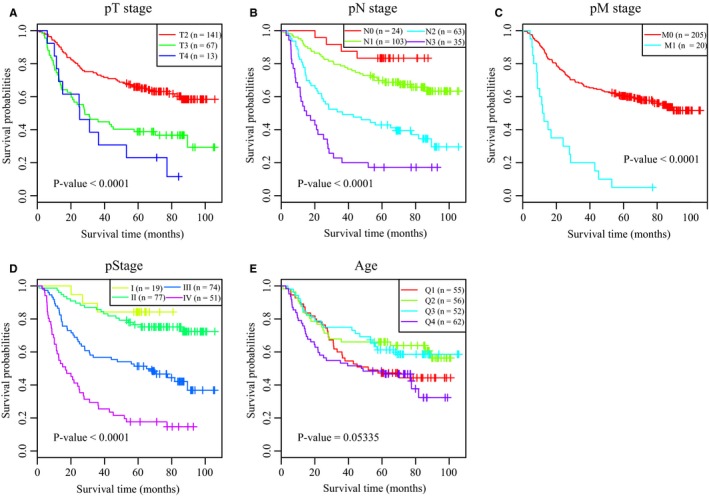
Screening of prognosis‐related clinical characteristics by Kaplan‐Meier analyses. A, Kaplan‐Meier curves based on different pT stages. B, Kaplan‐Meier curves based on different pN stages. C, Kaplan‐Meier curves based on different pM stages. D, Kaplan‐Meier curves based on different tumor stages. E, Kaplan‐Meier curves based on different age groups, where Q1, Q2, Q3, and Q4 represent quartiles

Taking into account the clinicopathological characteristics, we evaluated tumor invasion (pT), lymph node (pN), metastasis (pM), and tumor stage (pStage) using Kaplan‐Meier analysis. As shown in Figure 2, we found that all of the above clinical factors have a significant impact on the prognosis of GC patients (with a log‐rank test *P*‐value less than .0001). We further performed multivariate Cox proportional hazards modeling of each significant lncRNA probe (n = 177) combined with clinical characteristics as covariates. Thus, we performed 177 multivariate Cox analyses and finally a total of 46 lncRNA probes had a significance threshold < 0.01 (Table S5).

### 3.3 Establishment of a 6‐lncRNA signature by robust likelihood‐based survival and LASSO models

**Figure 3:**
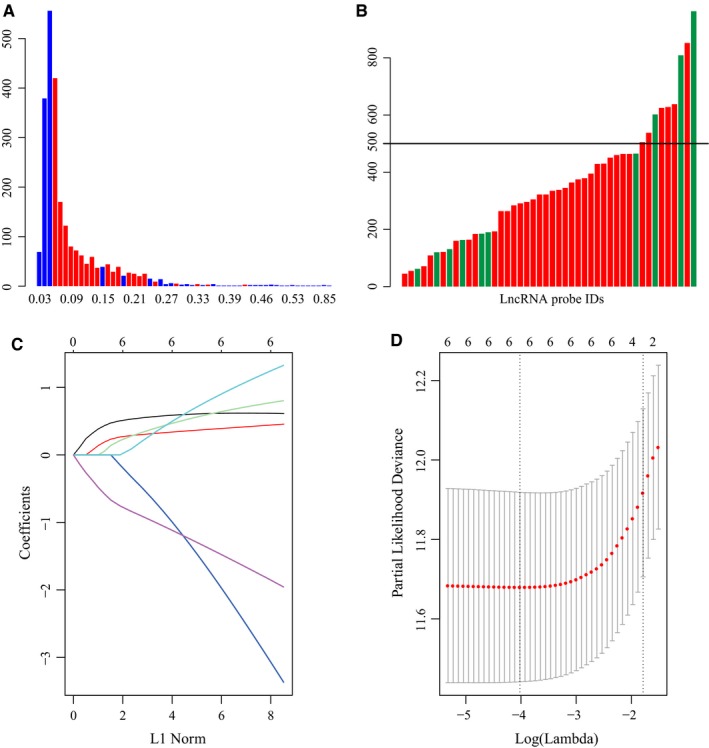
Screening of significant lncRNAs by robust likelihood‐based survival and LASSO models. A, The distribution of all lncRNA probes and standard deviation. The red bar indicates the standard deviation of the lncRNA probe with a frequency greater than 100; the horizontal axis represents the standard deviation, and the vertical axis represents the number of probes. B, The frequency distribution of lncRNA probes selected by the robust likelihood‐based survival model. The horizontal axis represents lncRNA probes, and the vertical axis represents the frequency of occurrence 1000 times. The red bar indicates the standard deviation of the lncRNA probe greater than the median standard deviation of all probes. C, Three‐time cross‐validation for tuning parameter selection in the LASSO model. D, The distribution of each lambda and CI

To identify lncRNAs related to survival, we used the R package *rbsurv* to construct a robust likelihood‐based survival model. It used the partial likelihood of the Cox model, which has been the basic method. The method implemented robust likelihood‐based survival analysis, repeated 1000 times. We calculated the standard deviation of all lncRNA probes (Figure 3A). The red bar represents lncRNA probes among the top 46 prognostic lncRNAs with frequencies >100. Finally, we selected lncRNAs with standard deviations greater than the standard deviations of all probes and frequencies greater than 500. As shown in Figure 3B, we identified a total of six lncRNA probes in our analysis, including 213447_at, 227909_at, 231925_at, 232191_at, 243017_at, and 244553_at.

Next, we performed a LASSO analysis, which selected lncRNAs affecting GC prognosis via regression coefficient shrinkage based on a penalty that is proportional to size. As shown in Figure 3C, we found that as the lambda increases, the number of independent coefficients tends toward zero. We used threefold cross‐validation to build this model. The confidence interval (CI) under each lambda was analyzed as shown in Figure 3D. The model was optimal when the lambda was 0.017953. Therefore, we chose the model with a lambda of 0.017953 as the final model, containing a total of six lncRNA probes. The prognostic score was imputed below: Risk score = 0.617 * IPW (lncRNA‐IPW) expression level + 0.429 * NCRNA00086 expression level + 0.744 * RP11‐38P22.2 expression level + (−2.794) * ERVH48‐1 expression level + 1.165 * LOC158572 expression level + (−1.761) * AC004080.17 expression level. The results of univariate Cox analysis and their details are shown in Table 3.

**Table 3:** The results of univariate Cox analysis and their information

Probe IDs	*P*‐value	HR	Low 95% CI	High 95% CI	Ref seq symbol	Ensembl symbol
213447_at	2.12E‐07	4.327	2.488	7.525	IPW	—
227909_at	4.17E‐06	4.957	2.507	9.801	NCRNA00086	NCRNA00086
231925_at	5.73E‐05	4.770	2.228	10.210	—	RP11‐38P22.2
232191_at	0.0026	0.009	0.000	0.191	—	ERVH48‐1
243017_at	0.0006	44.374	5.016	392.582	LOC158572	—
244553_at	1.33E‐05	0.070	0.021	0.232	—	AC004080.17

Abbreviations: CI: confidence interval; HR: hazard ratio; IPW, lncRNA‐IPW.

### 3.4 Prognostic evaluation by the 6‐lncRNA signature in GC

**Figure 4:**
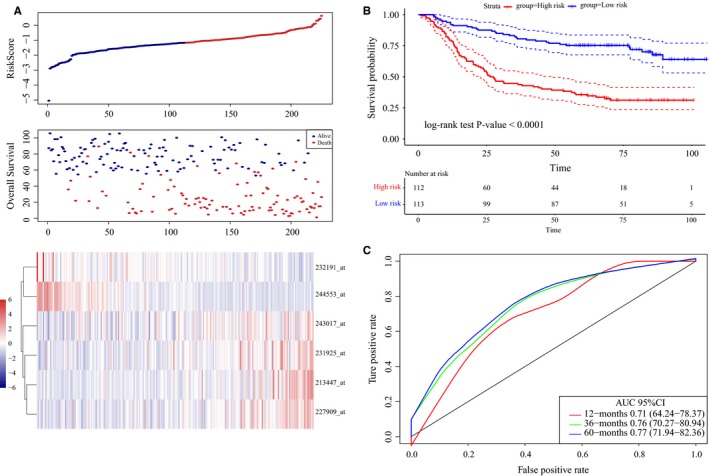
LncRNA risk score analysis using the training set http://www.ncbi.nlm.nih.gov/geo/query/acc.cgi?acc=GSE62254. A, Distribution of 6‐lncRNA‐based risk scores, lncRNA expression levels, and patient survival durations in the training set http://www.ncbi.nlm.nih.gov/geo/query/acc.cgi?acc=GSE62254 (n = 225). B, Kaplan‐Meier curves of OS according to the 6‐lncRNA signature. C, ROC curve analyses based on the 6‐lncRNA signature

The 6‐lncRNA signature‐based risk score for each patient was calculated in the training set (n = 225). The median cutoff point of risk scores was used to divide all patients into groups with a high risk (n = 112) and a low risk (n = 113). Figure 4A displays the distribution of the risk score for each patient in the training set. Patients with a high‐risk score had an obviously worse OS than patients with a low‐risk score (log‐rank test *P*‐value <.0001, Figure 4B). The probes 213447_at, 227909_at, 231925_at, and 243017_at with high expression levels and high‐risk scores were considered risk factors. In addition, the high expression levels of 232191_at and 244553_at and low‐risk scores were protective factors.

Additionally, ROC curve analyses were implemented to compare whether the survival prediction was sensitive and specific among the 6‐lncRNA signature‐based risk scores (Figure 4C). The AUCs were assessed by 1‐year (AUC = 0.71), 3‐year (AUC = 0.76), and 5‐year (AUC = 0.77) survival, suggesting that this 6‐lncRNA signature can effectively make the prognosis evaluation of GC patients.

### 3.5 The 6‐lncRNA signature as an independent factor for prognosis

The role of the 6‐lncRNA signature as an independent factor for GC prognosis was also tested. A univariate Cox regression analysis was implemented at first to determine the association between the 6‐lncRNA signature‐based risk score (HR = 3.05, 95% CI 2.26‐4.12, *P*‐value <.0001) and prognosis of GC (Table S6).

**Figure 5:**
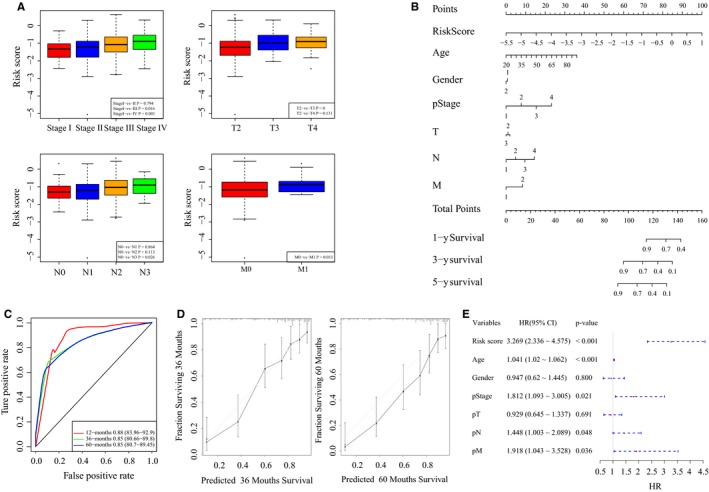
The association between the 6‐lncRNA signature and clinical characteristics. A, The distribution of risk scores according to different clinical information. B, The nomogram to predict the probabilities 1‐y, 3‐y, and 5‐y OS in patients. C, ROC curves according to the nomogram and lncRNA risk score. D, Calibration plots to predict the 3‐y and 5‐y OS of patients. The probability of survival predicted by the nomogram was plotted on the x‐axis, and actual survival was plotted on the y‐axis. E, The forest plot of risk scores and clinical characteristics

Furthermore, we analyzed the risk score distribution among different clinical stages, degrees of tumor invasion, degrees of lymph node involvement, and metastasis (Figure 5A). The results suggested that there were significant differences in risk scores based on different stages, and more advanced stages were associated with higher risk scores. Moreover, similar trends were observed for other clinical characteristics including pT, pN, and pM. Besides, we observed the prognostic evaluation ability of this model especially in Stage III and Stage IV in GC. As shown in Figure S1A,B, we found this 6‐lncRNA signature was with an AUC of 0.81 for 5‐year survival and the OS times of patients in the high‐risk group were significantly shorter than those in the low‐risk group in Stage III (log‐rank test *P*‐value <.0001). In addition, the 6‐lncRNA signature was with an AUC of 0.75 for 5‐year survival and different survival outcomes in Stage IV (log‐rank test *P*‐value = .02).

Next, we established a nomogram using clinical features, including age, sex, pT, pN, pM, pStage, and risk score (Figure 5B). The AUC for 3‐year survival using the predictive nomogram reached 0.85 (Figure 5C). According to the calibration curve, predictive values were consistent with observed values considering the probabilities of 3‐year OS and 5‐year OS (Figure 5D). Moreover, we used a forest plot to visualize the distribution of the clinical features including pT, pN, pM, pStage, and risk score by a multivariable Cox regression analysis (Figure 5E). The HR of the risk score was approximately 3.27 with a *P*‐value <.001 (Table S7).

### 3.6 Relationships between the 6‐lncRNA signature and biological functions

To observe the relationships between risk scores and biological functions across different samples, we selected the gene expression profiles corresponding to these samples using the R package *GSVA* for ssGSEA. By calculating the scores for each sample based on different biological functions, we further calculated the correlation between these functions and risk scores (Table S8). As shown in Figure S2A, Kyoto Encyclopedia of Genes and Genomes (KEGG) pathways with correlations greater than 0.3 were selected. Most of these pathways were negatively correlated with the sample risk scores, and a small number of pathways were positively related to the risk score. We selected the top 20 most relevant KEGG pathways and performed cluster analysis based on their enrichment scores (Figure S2B). Pathways such as adherens junction, gap junction, and Wnt signaling were activated as the risk score increased. Moreover, pathways such as the p53 signaling and base excision repair were suppressed as the risk score increased, suggesting an imbalance in these pathways in GC.

### 3.7 Validation of the 6‐lncRNA signature for prognostic evaluation

**Figure 6:**
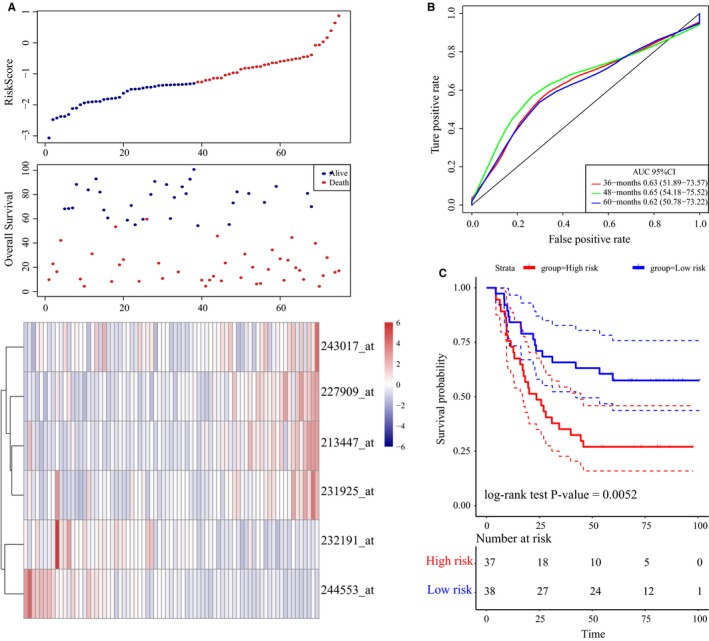
LncRNA risk score analysis using the internal validation set http://www.ncbi.nlm.nih.gov/geo/query/acc.cgi?acc=GSE62254. A, Distribution of 6‐lncRNA‐based risk scores, lncRNA expression levels and patient survival durations in the internal validation set http://www.ncbi.nlm.nih.gov/geo/query/acc.cgi?acc=GSE62254 (n = 75). B, ROC curve analyses based on the 6‐lncRNA signature. C, Kaplan‐Meier curves of OS based on the 6‐lncRNA signature

To determine the robustness of this model, we used the same coefficients in the validation sets. First, using the same risk formula in the internal validation set http://www.ncbi.nlm.nih.gov/geo/query/acc.cgi?acc=GSE62254 (n = 75), we classified patients into groups with a high risk (n = 37) and a low risk (n = 38) taking the median score as the cutoff point. The distributions of risk scores, survival durations of patients, and lncRNA expression levels are shown in Figure 6A. The Kaplan‐Meier curves of OS suggested that patients with high‐risk scores had significantly worse OS than patients with low‐risk scores (log‐rank test *P*‐value = .005, Figure 6C). The AUC exhibited by the 6‐lncRNA signature for 5‐year survival reached 0.65 (Figure 6B).

**Figure 7:**
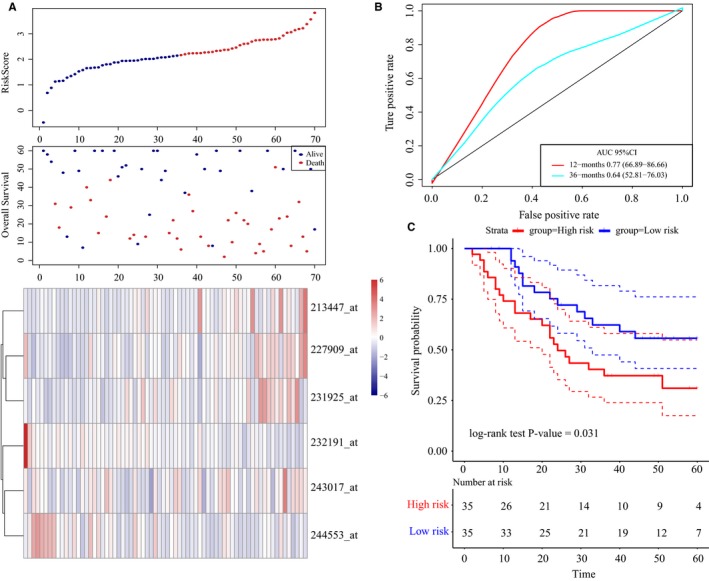
LncRNA risk score analysis using the external validation set http://www.ncbi.nlm.nih.gov/geo/query/acc.cgi?acc=GSE57303. A, Distribution of 6‐lncRNA‐based risk scores, lncRNA expression levels, and patient survival durations in the external validation set http://www.ncbi.nlm.nih.gov/geo/query/acc.cgi?acc=GSE57303 (n = 70). B, ROC curve analyses based on the 6‐lncRNA signature. C, Kaplan‐Meier curves of OS based on the 6‐lncRNA signature

In addition, the distribution of risk scores, lncRNA expression signatures, and survival durations in http://www.ncbi.nlm.nih.gov/geo/query/acc.cgi?acc=GSE62254 (n = 300) was evaluated (Figure S3A). We also validated the robustness of this 6‐lncRNA signature with an AUC of 0.73 for 5‐year survival (Figure S3B). Moreover, the survival outcomes of patients in the high‐risk group were worse than that of patients in the low‐risk group (log‐rank test *P*‐value <.0001, Figure S3C). In agreement with the abovementioned findings, the OS times of patients in the high‐risk group were markedly shorter than that of patients in the low‐risk group from the external validation set (http://www.ncbi.nlm.nih.gov/geo/query/acc.cgi?acc=GSE57303, n = 70, log‐rank test *P*‐value = 0.03, Figure 7A‐C). According to the abovementioned findings, the 6‐lncRNA signature can be used to effectively make the prognosis evaluation of GC patients.

## 4 DISCUSSION

In this study, we performed a multistep re‐annotation analysis of lncRNA expression in GC. Based on the lncRNA expression profiles of http://www.ncbi.nlm.nih.gov/geo/query/acc.cgi?acc=GSE62254, robust likelihood‐based survival and LASSO models were used to establish a 6‐lncRNA signature with prognostic value. In addition, the internal validation set http://www.ncbi.nlm.nih.gov/geo/query/acc.cgi?acc=GSE62254 and the external validation set http://www.ncbi.nlm.nih.gov/geo/query/acc.cgi?acc=GSE57303 were successfully used to validate the robustness of our 6‐lncRNA signature. The above results indicated that the 6‐lncRNA signature exhibited a robust ability to make the prognosis evaluation of GC patients.

Prognostic evaluation remains necessary for the selection of appropriate treatments for cancer patients because of poor prognosis. Recently, lncRNAs have been identified as significant regulators in various human cancers. Moreover, some lncRNAs and lncRNA signatures have been treated as potential prognostic indicators. Based on a meta‐analysis, lncRNA BLACAT1 may serve as a prognostic predictor in cancer,^16^ similar to other well‐known lncRNAs, such as H19,^15^ PVT1,^31^ HOTAIR,^32^ and PANDAR.^33^ There have been many studies on the lncRNA signatures for GC. For example, Zhu et al^34^ used http://www.ncbi.nlm.nih.gov/geo/query/acc.cgi?acc=GSE62254 and http://www.ncbi.nlm.nih.gov/geo/query/acc.cgi?acc=GSE15459 datasets to establish a set of 24 lncRNAs that showed an obvious relation to disease‐free survival (DFS) in GC. In addition, based on the TCGA database and LASSO models, Cheng identified a 3‐lncRNA prognostic signature including RP11‐108 M12.3, CYP4A22‐AS1, and AP000695.6.^35^ Using the random survival forests method, Song et al^36^ found a set of three lncRNAs including TGFB2‐OT1, LINC01140, and RP11‐347C12.10. Fan et al^37^ also used GEO datasets and survival forest algorithm to build a 5‐lncRNA signature with prognostic value of 0.86. In another study,^38^ http://www.ncbi.nlm.nih.gov/geo/query/acc.cgi?acc=GSE79973, including 10 paired GC and normal tissues, was first used to identify the differentially expressed lncRNAs. Based on LASSO Cox regression model, 12‐lncRNA signature was finally identified with AUC of 0.869. Although there have been a series of methods for lncRNA signature establishment, the power of prognostic evaluation was different for each study. The ROC values of some models proposed in other literatures were higher than the model proposed in our study; however, the number of lncRNAs in these prognostic signatures was much higher than that in our 6‐lncRNA signature (5‐year AUC = 0.77 in our study). Considering clinical application values, the number of lncRNAs in the model should be as small as possible with high prognostic evaluation value. Here, we compared the predictive power using the AUC value among recent reports on lncRNA signatures in GC (Table 4).

**Table 4:** The comparison of studies about lncRNA signature for GC

Databases	Methods	LncRNA signature	LncRNA symbols	AUC value	Reference
http://www.ncbi.nlm.nih.gov/geo/query/acc.cgi?acc=GSE62254 and http://www.ncbi.nlm.nih.gov/geo/query/acc.cgi?acc=GSE15459	Random survival forest‐variable hunting	24‐lncRNAs	AF035291, AI028608, AK026189, H04858, BC037827, BC038210, AI916498, AA463827, AA041523, BE621082, AK056852, AW206234, AL703532, AI095542, AI080288, BC021187, BF238392, BC005107, BC039674, AI056187, T79746, H11436, BF511694, and BC035722	0.82	Zhu et al
TCGA	LASSO Cox regression model	3‐lncRNAs	CYP4A22‐AS1, AP000695.6, and RP11‐108M12.3	0.737	Cheng et al
http://www.ncbi.nlm.nih.gov/geo/query/acc.cgi?acc=GSE62254 and http://www.ncbi.nlm.nih.gov/geo/query/acc.cgi?acc=GSE15459	Univariable Cox regression analysis and random survival forest‐variable hunting	3‐lncRNAs	LINC01140, TGFB2‐OT1, and RP11‐347C12.10	0.688	Song et al
TCGA	Limma, univariate, and multivariate Cox regression models	5‐lncRNAs	CTD‐2616J11.14, RP1‐90G24.10, RP11‐150O12.3, RP11‐1149O23.2, and MLK7‐AS1	None	Ren et al
http://www.ncbi.nlm.nih.gov/geo/query/acc.cgi?acc=GSE65801, http://www.ncbi.nlm.nih.gov/geo/query/acc.cgi?acc=GSE29998, E‐MTAB‐1338, and TCGA	Weighted correlation network and LASSO analysis	11‐lncRNAs	ARHGAP5‐AS1, FLVCR1‐AS1, H19, HOTAIR, LINC00221, MCF2L‐AS1, MUC2, PRSS30P, SCARNA9, TP53TG1, and XIST	None	Zhang et al
http://www.ncbi.nlm.nih.gov/geo/query/acc.cgi?acc=GSE27342, http://www.ncbi.nlm.nih.gov/geo/query/acc.cgi?acc=GSE38749, http://www.ncbi.nlm.nih.gov/geo/query/acc.cgi?acc=GSE50710, and http://www.ncbi.nlm.nih.gov/geo/query/acc.cgi?acc=GSE63089	Random survival forest‐variable hunting	5‐lncRNAs	AK001094, AK024171, AK093735, BC003519, and NR_003573	0.95	Fan et al

Robust likelihood‐based survival modeling is frequently used in prognostic signature construction for cancers in recent years. For example, a prognostic 11‐lncRNA expression signature was constructed for breast invasive carcinoma (BRCA).^39^ In that study, He et al used the lncRNA expression profiles of BRCA samples obtained from the TCGA database. They carried out a univariable Cox analysis for the convenience of primary screening and repeated the modeling process approximately 1000 times for robust likelihood‐based survival. Thus, 11 lncRNAs with frequencies exceeding 600 were chosen for predicting BRCA prognosis, which offered new insights into the potential treatment approaches for breast cancer. For lung squamous cell carcinoma, Luo et al^40^ used RNA‐Seq data of primary lung cancer samples from TCGA database and a robust likelihood‐based survival model to establish a 4‐lncRNA‐based prognostic model. In addition, a 4‐lncRNA prognostic signature was identified in head and neck squamous cell carcinoma by Diao et al^41^ based on TCGA database using robust likelihood‐based survival, random sampling iteration, and univariate Cox regression survival analyses. However, there have been no studies on lncRNA signatures in GC.

In our study, using the robust likelihood‐based survival and LASSO models, we established a 6‐lncRNA signature with prognostic value in GC. These six lncRNAs were IPW, NCRNA00086, RP11‐38P22.2, ERVH48‐1, LOC158572, and AC004080.17. The human homologue IPW of the lncRNA IPW is located on chromosome 15, which is deleted in over 70% of patients with Prader‐Willi syndrome (PWS).^42^ ERVH48‐1 has been considered as a new biomarker for evaluation of the prognosis of tongue squamous cell carcinoma by analyzing the competing endogenous RNA network associated with lncRNAs.^43^ However, NCRNA00086, RP11‐38P22.2, LOC158572, and AC004080.17 have been reported for the first time in our present study. According to these findings, these lncRNAs might play an unknown biological role in GC tumorigenesis.

Regarding the results of ssGSEA, we found that several pathways, such as adherens junction, gap junction, calcium signaling, actin cytoskeleton regulation, extracellular matrix receptor interaction, Wnt signaling, and mammalian target of rapamycin (mTOR) signaling, were positively associated with risk scores. The mTOR signaling pathway participates in different human cancers.^44^ Oncogenic activation of the mTOR signaling pathway is beneficial for the development, proliferation, and survival of cancer cells, which emphasizes that targeting carcinogenic mTOR pathway members can be used to effectively treat cancer.^45^ In addition, catenins and cadherins act as central molecules between cells in adherens junction and play essential roles in cell adhesion, tissue morphogenesis, and cancer.^46^ Therefore, the activation of the above pathways may lead to tumorigenesis and the development and progression of GC.

Accurate prognosis is the basis for planning appropriate treatments for cancer patients. Due to the heterogeneity, the stage of greater change in survival rate will encounter greater uncertainty. Patient outcomes vary widely even within similarly staged cohorts.^47^ Thus, techniques utilizing multidimensional data not limited to tumor, node, metastasis staging system with histopathological features can improve the prognosis of cancer patients. For example, Dimitriou et al^47^ made a point that a principled machine learning framework can improve accuracy of stage II colorectal cancer prognosis. In our study, we identified a 6‐lncRNA prognostic signature in GC. Besides, we observed the prognostic evaluation ability of this model especially in Stage III and Stage IV in GC. Results determined that our model had the value of prognostic evaluations in patients with advanced GC.

Although the 6‐lncRNA prognostic signature identified in our study was robust, there were several limitations. First, we need to validate this 6‐lncRNA signature in large‐scale clinical GC samples. Second, this signature should be subjected to prospective validation prior to its clinical applications. Finally, whether the 6‐lncRNA combined with other clinical characteristics can increase the predictive power of based on AUC values remains an interesting question for us.

In conclusion, we performed multistep prognostic analyses of lncRNAs in GC. Robust likelihood‐based survival and LASSO models were used to successfully establish a 6‐lncRNA prognostic signature. The robustness of our 6‐lncRNA signature was also validated. In conclusion, the 6‐lncRNA signature can effectively make GC patient prognosis evaluation.

## CONFLICT OF INTEREST

The authors declare that there was no conflict of interest.

## AUTHORS' CONTRIBUTIONS

BM conceived and designed the study. BM and YML performed this work. BM drafted the manuscript. YPR reviewed and revised the paper. All authors read and approved the manuscript.

## Data Availability

The data used for supporting the results of the study are included within the article.
