# Supplementary material for: Identification of a 6‐lncRNA prognostic signature based on microarray re‐annotation in gastric cancer
Source: Cancer Med. 2019 Nov 19;9(1):335–49. doi: 10.1002/cam4.2621 (PMC6943089; doi:10.1002/cam4.2621)
Supplement: Supplementary file 12 [file CAM4-9-335-s012.docx]

**Supplementary Information**

**Supplementary Table Legends**

**Supplementary Table 1.** The clinical information of GSE62254 (n = 300).

**Supplementary Table 2.** The clinical information of GSE57303 (n = 70).

**Supplementary Table 3.** Re-annotation of lncRNA classification based on NetAffx Annotation Files.

**Supplementary Table 4.** The significant lncRNA probes by univariate Cox proportional hazard model.

**Supplementary Table 5.** The significant lncRNA probes by multivariate Cox proportional hazard model.

**Supplementary Table 6.** Univariate Cox regression analysis of the six-lncRNA risk score and clinical information.

**Supplementary Table 7.** Multivariable Cox regression analysis of the six-lncRNA risk score and clinical information.

**Supplementary Table 8.** Relationships between the six-lncRNA signature and biological functions by ssGSEA analysis.

**Supplementary Figure Legends**

**Supplementary Figure 1.** **The prognostic evaluation value of this model in Stage III and Stage IV in GC.** (A) The ROC analysis based on the six-lncRNA signature in Stage III. (B) The Kaplan-Meier curve of OS based on the six-lncRNA signature in Stage III. (C) The ROC analysis based on the six-lncRNA signature in Stage IV. (D) The Kaplan-Meier curve of OS based on the six-lncRNA signature in Stage IV.

**Supplementary Figure 2. Relationships between the six-lncRNA signature and biological functions by ssGSEA analysis.** (A) The correlation heatmap of KEGG pathways with correlation greater than 0.3. (B) The top 20 most relevant KEGG pathways. The horizontal axis represents the samples. As the risk score increases, the left-to-right risk score increases.

**Supplementary Figure 3.** **LncRNA risk score analysis of entire GSE62254 set.** (A) The distribution of six-lncRNA risk score, patients’ survival status and lncRNA expression signature in entire GSE62254 set (n = 300). (B) The ROC analysis based on the six-lncRNA signature. (C) The Kaplan-Meier curve of OS based on the six-lncRNA signature.
